# Uncertainty of Object Points Monoplotted from Terrestrial Images

**DOI:** 10.1007/s41064-025-00359-6

**Published:** 2025-10-13

**Authors:** Sebastian Mikolka-Flöry, Camillo Ressl, Norbert Pfeifer

**Affiliations:** TU Wien, Department of Geodesy and Geoinformation, Research Unit Photogrammetry, Vienna, Austria

**Keywords:** Monoplotting, Terrestrial images, Monte carlo simulation, Variance propagation, Uncertainty estimation, Unscented transform, Silhouette detection

## Abstract

With monoplotting, object points can be reconstructed from a single oriented image if a reference surface of the captured scene is available. While used extensively in environmental sciences, prior approaches fall short of describing the uncertainty of the reconstructed points.

In this paper, we estimate this monoplotting uncertainty using three different methods: i) Monte Carlo simulation, ii) unscented transform and iii) classical variance propagation with tangential approximation of the terrain. Our investigations are guided by two different use cases: i) For manually selected image points, the estimated uncertainty determines whether these monoplotted points are accurate enough for a subsequent research question (e.g. deriving glacier changes from historical terrestrial images). ii) Estimating the monoplotting uncertainty for each pixel of the whole image to get an overview of the expectable uncertainty, which will already be beneficial during the image orientation step. While for the first use case, the precision of the estimated uncertainty is crucial, the second use case requires a fast method. Furthermore, in both use cases, silhouettes must be considered because the estimates in their vicinity will not be valid. Therefore, we further investigate the derivation of silhouette masks, optimally exploiting the available information from the three different methods.

For evaluation, we use a selected historical terrestrial image showing a glacier in the Alps around 1900, where, for the first use case, we manually digitised individual vertices of a glacier outline. Using the Monte Carlo estimates based on 1000 samples as reference, the results from the unscented transform are closer to those (14.1% RMS) than the ones from variance propagation (24.7% RMS). Despite this good result from the unscented transform, our recommendation for this use case is nevertheless the Monte Carlo simulation, thanks to the speed of existing ray-casting routines.

However, for the second use case, where the monoplotting uncertainty is predicted for each pixel of the entire image to get a quick overview, the enormous amount of millions of ray-castings prohibits both Monte Carlo simulation and unscented transform. Here, we propose to use variance propagation because of its speed and still reasonable precision, yielding uncertainty estimates with an RMS of 7.8% in areas away from silhouettes.

## Introduction

Single terrestrial images taken as memories by locals and tourists are a unique resource to document long-term changes (e.g. glacier retreat) in alpine regions. Historical terrestrial images, which have been available since the 1890s and thus predate other historical resources (e.g. aerial nadir images) for up to 50 years, are of great value for environmental sciences. However, as such historical terrestrial images were typically acquired without any stereo intention and have large time gaps in between, deriving 3D information from overlapping images based on Structure from Motion is difficult and often not possible. Hence, monoplotting poses an interesting alternative to derive metric information.

**Fig. 1:**
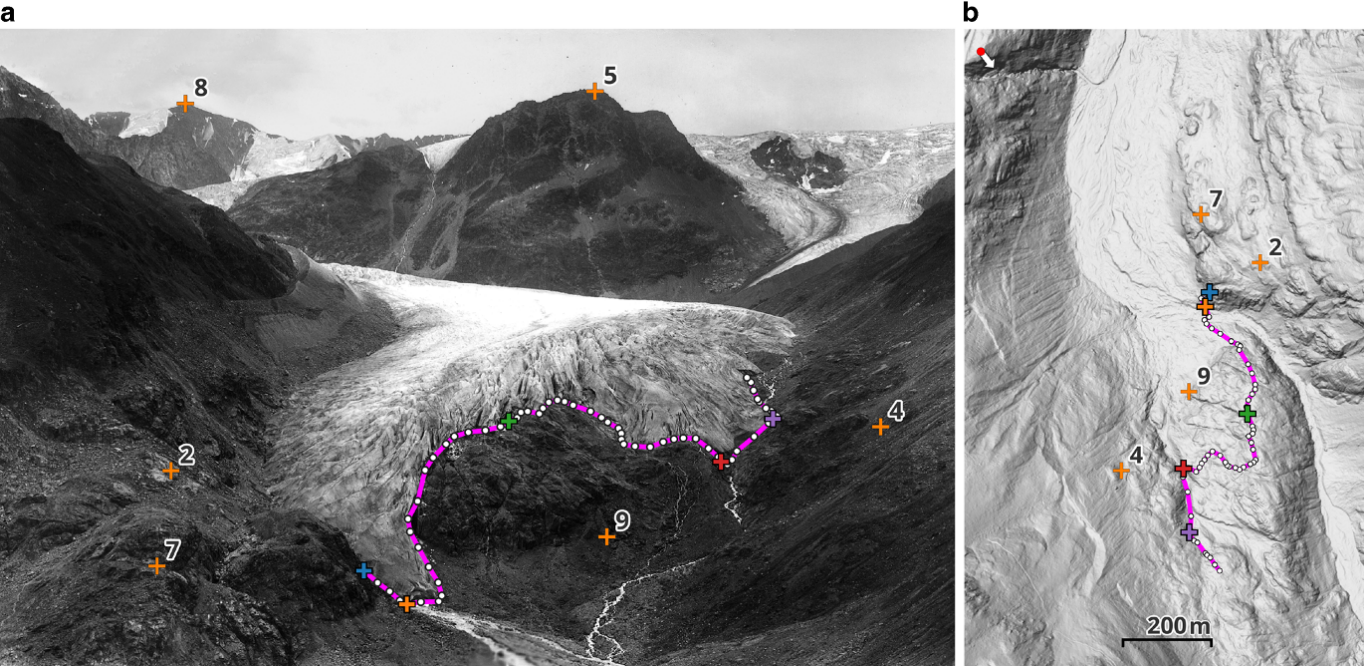
Documenting the past glacier extent of the Gepatschferner in Austria using a historical terrestrial image from around 1900, provided by Martin Frey. None of the GCPs is close to the glacier outline, neither in image nor object space. Thus, the GCP residuals tell little about the monoplotting uncertainty of the vertices of this outline. **a** Historical terrestrial image with GCPs and glacier outline (purple) in image space. **b** Selected GCPs and monoplotted glacier outline displayed in object space on top of a recent hillshade

Monoplotting works by connecting a selected image point with the projection centre and intersecting this image ray with a digital terrain model (DTM). The resulting object point is unique, provided it does not lie on a silhouette. Thus, in general, coordinates in object space are directly derived from image points. This way, linear geometries like glacier outlines can be represented as polylines in object space by monoplotting each line vertex, see Fig. 1.

Widely utilised in various earth science disciplines to quantify long-term landscape changes from single images (Altmann et al. 2023; Gabellieri and Watkins 2019; Genuite et al. 2023; Marco et al. 2018; Wiesmann et al. 2012; Bühler et al. 2019), subsequent spatial analysis should take the uncertainty of the monoplotted points into account, i.e. the covariance matrix of each point or statistical measures derived from it. This way, one can determine whether the observed changes are actually larger than this uncertainty. However, in none of these previous works, this uncertainty is properly addressed: It is either not mentioned at all, determined through comparison with reference data (Bayr 2021) or limited to the ground control points (GCPs) (Stockdale et al. 2015). Suitable reference datasets are often unavailable or highly uncertain, especially for historical images. Analysis based on GCPs appears reasonable as they are used for the spatial resectioning of each single image. Yet, GCPs are generally identified along prominent topographic structures (e.g. horizon, ridgelines) that are rarely close to the features of interest (Fig. 1). Due to the changing geometric relations within the view (distances and intersection angles), the monoplotting uncertainty varies significantly within an image. Consequently, the residual measures available at the individual GCPs are, in most cases, not representative of the monoplotted points of interest, and instead, an actual uncertainty measure is needed for them.

Another potential use case for the monoplotting uncertainty is related to the image orientation itself. GCPs for orienting historical images are typically identified manually in a recent orthophoto and a DTM. Since this can be quite a challenging and time-consuming process, at some point, the user is confronted with the question of whether the achievable monoplotting accuracy is sufficient or if additional GCPs are needed. It is difficult to answer this solely from the variances of the estimated camera parameters, due to the combined influence of acquisition geometry and observed topography. Hence, predicting the monoplotting uncertainty for *each* pixel of the image, further referred to as the uncertainty map, can serve as a valuable tool during the whole image orientation workflow.

The method for estimating the monoplotting uncertainty should therefore be both accurate and fast (considering that the uncertainty map scenario requires the method to be executed for every pixel of an image).

A direct uncertainty formula offers the chance for a fast solution. However, given the irregular shape of the DTM, such a direct solution is not easy to come by. For the specific case of the object being a plane, the problem of ray-plane intersection is solved in (Förstner and Wrobel 2016, p. 527) by classical variance propagation using first-order terms with respect to the image ray geometry. Applying this direct solution on a real terrain requires the area around the monoplotted object point to be approximated by its tangent plane. The validity of this first-order approximation (now also with respect to the terrain) depends on the local terrain neighbourhood (e.g. smoothness, curvature) and, consequently, impacts the accuracy of the estimated monoplotting uncertainty. To avoid this approximation, alternatives are required.

One method, which requires no first-order approximation at all and takes into account the actual shape of the terrain around the monoplotted point, is Monte Carlo simulation (Metropolis and Ulam 1949). Thereby, many *randomly* selected variations of the ray are intersected with the actual terrain. This method is controllable with respect to the precision of the estimates. Depending on the desired precision, a large number of random samples, e.g. several hundred, may be necessary. This, in turn, makes Monte Carlo simulation not appear as a promising method for deriving the uncertainty map.

An efficient and accurate alternative to Monte Carlo simulation is the unscented transform (Julier and Uhlmann 1997), which only needs a small number of *systematically* selected rays. The downside is that it requires a scaling parameter. Its choice is up to the user and may result in illegal results (i.e., negative-definite estimates for the covariance matrix).

Despite their pros and cons regarding precision and run time, all three mentioned methods appear as viable candidates for the uncertainty estimation. In our work, we consider two practical use cases for which we want to discuss the estimation of the monoplotting uncertainty:

1. for individual, manually identified, points of interest (e.g. vertices of a polyline): Here, the precision of the uncertainty estimate is more important than the computation speed. Thus, both Monte Carlo simulation and unscented transform appear as candidates, although the much larger number of samples for Monte Carlo makes unscented transform appear to be more promising.
2. for each pixel of the image (uncertainty map): Here, the computation speed will be of concern, and even the unscented transform might take too long. Thus, the first-order approximation, which offers a direct solution without any samples, appears as a reasonable candidate.

In both use cases, silhouettes, which separate the visible terrain from the obstructed terrain, need to be considered. For the methods based on ray sampling (Monte Carlo simulation, unscented transform), silhouettes lead to a multimodal distribution of the monoplotted sample points. Hence, the derived “variances” cannot be interpreted as uncertainties. On the other hand, the variance propagation based on the local tangent plane does not capture the depth discontinuities, and therefore, the estimated variances will be wrong. Thus, it is essential to take care of the silhouettes in all three approaches and either exclude the area around them by masking or warn the user. Since different intermediate results arise during the three uncertainty estimation approaches, we adapted the silhouette detection for each approach to exploit these intermediate results optimally and avoid extensive unnecessary calculations.

Accordingly, the contributions of this paper are the following:

- Discussion of different approaches for deriving the monoplotting uncertainty: i) Monte Carlo simulation, ii) unscented transform, and iii) first-order variance propagation.
- Derivation and evaluation of silhouette masks.
- Demonstrating the two considered use cases on a historical terrestrial image. Thereby evaluating the methods with respect to accuracy and speed. Since the precision of Monte Carlo is controllable, it will be used to generate the reference values for this experimental evaluation.

The paper is structured in the following way: We first introduce the factors influencing the monoplotting uncertainty (Sect. 2) and, based on these considerations, we explain the employed methods in Sect. 3. The description of the dataset used in our experiments is given in Sect. 4. The experiments and the obtained results make up Sect. 5, followed by their discussion in Sect. 6. Finally, a conclusion in Sect. 7 ends the paper.

## Factors Influencing the Monoplotting Uncertainty

As mentioned above, we formulate monoplotting as the intersection of rays originating from the projection centre of a camera with a DTM. Therefore, the uncertainty of the 3D points derived through monoplotting depends on the uncertainty of the camera pose, the image measurements and the DTM.

### Camera Pose

The orientation of a single image is computed using GCPs. Following (Förstner and Wrobel 2016, p. 501) this can be formulated in the six elements of the exterior orientation (projection centre ***Z*** and three rotation angles $$\alpha,\zeta,\kappa$$) and, if an unknown camera was used, like in our scenario of historical images, the three elements of the interior orientation ($$x_{0}, y_{0}, f)$$. Thus, the uncertainty of the camera pose is represented by the covariance of the orientation parameters as $$\mathsf{\Sigma}_{hh}$$ with $$\boldsymbol{h}^{\mathsf{T}} = (\boldsymbol{Z}^{\mathsf{T}}, \alpha, \zeta, \kappa, x_{0}, y_{0}, f)$$.

### Image Measurements

Various factors influence the measurement accuracy of image points, such as unmodelled systematic effects (e.g. distortion) or circumstances related to their identification (e.g. contrast, blur). Especially the latter change from pixel to pixel (e.g. contrast) and are user-specific (e.g. distinguishability of the boundary between semantic classes). We represent these image measurement uncertainties by the variance ${\sigma}_{m}^{2}$, which allows us to introduce them into the derivation of the monoplotting uncertainty.

### Digital Terrain Model

We use a recent DTM derived from Airborne Laser Scanning. With their high point density, vertical and positional accuracy (Aguilar et al. 2005; Vosselman 2008), we assume the influence of the uncertainty of the DTM on the monoplotting uncertainty to be minor. This is especially true for historical terrestrial images where, for example, the uncertainty of the unknown projection centre may reach several meters. Therefore, throughout this work, we assume the DTM to be error-free.

Of course, using a recent DTM to represent scenes captured over 100 years ago raises the question of whether its usage is justified and whether the area of interest can be considered stable over this extended time period. This influence could be addressed if multiple DTMs from different epochs are available. One possible way would be to evaluate the significance of change from a series of DTMs and their differences (Wheaton et al. 2010). This would result in a binary map indicating stable areas in the simplest case. Alternatively, monoplotting could be conducted for each DTM individually. The resulting differences in the derived object coordinates could then be integrated into the uncertainty estimation. However, accurate height information close to the acquisition date is usually unavailable, making it difficult to quantify the magnitude of topographic changes. Accordingly, monoplotting of historical images based on a current DTM should only be employed in areas where the topography can be assumed to be relatively stable compared to the observed changes.

Beyond the choice of the DTM, its representation also impacts the calculation of the monoplotting uncertainty. We use a piecewise planar triangulated mesh. This way, in the context of our work, monoplotting is always a ray-plane intersection, which has two explicit advantages: First, the monoplotting point can be calculated by ray casting using highly optimised libraries like Embree (Wald et al. 2014). Second, the intersected triangle can serve as the local tangent plane of the monoplotted point. This approximation is required to estimate the monoplotting uncertainty by classical variance propagation. A negative consequence of this piecewise planar triangulation is that the same local tangent plane will be utilised for each monoplotting point within a triangle, independent of its location within the triangle. This will distort the monoplotting uncertainty estimated by variance propagation for points close to a triangle edge—as opposed to the two ray sampling approaches, which actually consider the variation of the triangles.

## Method

After discussing the major factors influencing the uncertainty estimation of monoplotted points, we need to describe the relationship between the coordinates of corresponding points in image and object space. This relation can be formulated by the collinearity equation in a Euclidean vector form as

1 $$\lambda\textit{\textsf{R}}(\boldsymbol{x}_{p}-\boldsymbol{z}) = \boldsymbol{X}_{\!P}-\boldsymbol{Z}.$$

Here, $$\boldsymbol{x}_{p} = (x_{p}, y_{p}, 0)^{\mathsf{T}}$$ is the image point, $$\boldsymbol{X}_{\!P} = (X_{P}, Y_{P}, Z_{P})^{\mathsf{T}}$$ is the corresponding object point, $$\boldsymbol{z} = (x_{0}, y_{0}, f)^{\mathsf{T}}$$ is the interior orientation (with principal distance $$f > 0)$$, $$\boldsymbol{Z} = (X_{0}, Y_{0}, Z_{0})^{\mathsf{T}}$$ is the projection centre, and *R* is the rotation matrix of the image (the chosen parametrisation is detailed in Sec. 8). Image and object space are related by an unknown scale factor $$\lambda$$, which changes from point to point. The collinearity equation in Eq. 1 serves not only as basis for monoplotting, but also for the ray casting required for the two considered sample based methods (i.e. unscented transform and Monte Carlo simulation).

### Monoplotting as Ray-plane Intersection

Following the triangulation-based representation of the DTM, we can formulate monoplotting as a ray-plane intersection. The object point ***M***, obtained by monoplotting the image pixel ***m***, thus results from intersecting the image ray (from Eq. 1)

2 $$\boldsymbol{M} = \boldsymbol{Z} + \lambda\textit{\textsf{R}}(\boldsymbol{m}-\boldsymbol{z}) = \boldsymbol{Z} + \lambda\boldsymbol{d}$$

with the plane

3 $$\boldsymbol{n}^{\mathsf{T}} (\boldsymbol{M}-\boldsymbol{P}_{0}) = 0,$$

which is given by its normal vector ***n*** and some point $\boldsymbol{P}_{0}$. Combining these two equations gives the unknown scale factor as

4 $$\lambda = \dfrac{\boldsymbol{n}^{\mathsf{T}} (\boldsymbol{P}_{0}-\boldsymbol{Z})}{\boldsymbol{n}^{\mathsf{T}} \boldsymbol{d}},$$

which yields the intersection point ***M*** as

5 $$\boldsymbol{M} = \boldsymbol{Z} + \dfrac{\boldsymbol{n}^{\mathsf{T}} (\boldsymbol{P}_{0}-\boldsymbol{Z})}{\boldsymbol{n}^{\mathsf{T}} \boldsymbol{d}} \boldsymbol{d}.$$

Obviously, no intersection point can be derived if the direction vector ***d*** of the image ray is parallel to the plane; i.e. $\boldsymbol{n}^{\mathsf{T}} \boldsymbol{d} = 0$. In Eq. 5 all considered stochastic parameters (i.e. camera orientation parameters and image measurements) are contained and hence, it will serve as basis for the uncertainty estimation based on classical variance propagation.

### Estimation of the Monoplotting Uncertainty

With the collinearity equation Eq. 1 and the ray-plane intersection Eq. 5 we established the geometrical foundation for the required uncertainty estimation methods. Together with the uncertainty of the camera parameters and the image point as input, we now want to calculate the uncertainty of the monoplotting point ***M***. More formally, this can be described as the task of calculating the covariance matrix $\mathsf{\Sigma}_{M\!M}$ for the random variable $\boldsymbol{\underline{M}}$, which is a function $\boldsymbol{f}(\boldsymbol{\underline{x}})$ of the random variable ${\boldsymbol{\underline{x}} \sim \mathcal{N}(\boldsymbol{\mu}_{x}, \mathsf{\Sigma}_{xx})}$ for which we assume a normal distribution (Förstner and Wrobel 2016,. p. 42). In $\boldsymbol{\underline{x}}$ we combine the following 11 elements: The coordinates of the projection centre ***Z***, the Euler angles ($\alpha,\zeta,\kappa$), the coordinates of the principal point ($x_{0}, y_{0}$) and the principal distance (*f*) and the coordinates of the image point $(x_{m}, y_{m})$ to be monoplotted. Accordingly, the covariance matrix $\mathsf{\Sigma}_{xx}$ combines the fully occupied covariance matrix $\mathsf{\Sigma}_{hh}$ from the image orientation and two diagonal entries with the variance of the image measurements ($\sigma_{m}^{2}$).

As mentioned previously, we selected three different uncertainty estimation methods which appear as viable candidates to calculate $\mathsf{\Sigma}_{M\!M}$: Monte Carlo simulation and unscented transform, which are based on image ray samples, and classical variance propagation using first-order terms of the Taylor series based on Eq. 5. Those three approaches will be discussed in more detail in the following.

#### Monte Carlo Simulation

Monte Carlo simulation (Metropolis and Ulam 1949) is a well-established method for propagating uncertainties in complex systems (Anderson 1976; Zhang 2021). For the estimation of the monoplotting uncertainty, we draw *u* random samples of the uncertain image ray (by considering the covariance matrix $\mathsf{\Sigma}_{xx}$) and intersect them with the DTM. From the *u* intersection points, the covariance matrix $\mathsf{\Sigma}_{M\!M}$ of ***M*** can be calculated.

Usually, *u* is in the order of a few hundred to some thousands—depending on the anticipated accuracy. Assuming a normal distribution, the relative precision of an estimated standard deviation $\hat{\sigma}_{0}$ (Förstner and Wrobel 2016, p. 90) is calculated based on the redundancy *R* as

6 $$\frac{\sigma_{\hat{\sigma}_{0}}}{\hat{\sigma}_{0}} = \sqrt{\frac{1}{2R}}.$$

With Eq. 6, it is possible to estimate the required number of samples to achieve certain accuracies with the Monte Carlo simulation. For large sample sizes *u*, we have $R \sim u$, and with $u=1000$, the estimated standard deviations will be accurate to $\sim 2\%$, which suffices for the anticipated use cases and hence, will serve as reference throughout this work.

#### Unscented Transform

The unscented transform (Julier and Uhlmann 1997) is an efficient alternative to the Monte Carlo simulation. By *systematically selecting* $2n+1$ weighted points from a given *n*-dimensional distribution, referred to as sigma points, the mean and covariance of the transformed distribution can be approximated from the transformed set of sigma points.

Given an *n*-dimensional normally distributed random variable $\boldsymbol{\underline{x}} \sim \mathcal{N}(\boldsymbol{\mu}_{x}, \mathsf{\Sigma}_{xx})$, the $2n+1$ sigma points $\boldsymbol{x}_{0}, \dots, \boldsymbol{x}_{2n}$ are derived by

7 $$\boldsymbol{x}_{0} = \boldsymbol{\mu}_{x}$$

8 $$\boldsymbol{x}_{j} = \boldsymbol{\mu}_{x} + \sqrt{(n+\kappa)} \boldsymbol{s}_{j}$$

9 $$\boldsymbol{x}_{j+n} = \boldsymbol{\mu}_{x} - \sqrt{(n+\kappa)} \boldsymbol{s}_{j}$$

with $j=1, \dots, n$ and $\boldsymbol{s}_{j}$ being the rows or columns of the matrix square root of $\mathsf{\Sigma}_{xx}$. We used Cholesky decomposition as suggested by (Julier and Uhlmann 1997) because of its numerical stability and efficient calculation. The corresponding weights *w*_*i*_ are calculated as

10 $$w_{i} = \begin{cases} \frac{\kappa}{n+\kappa} & \text{if } i=0 \rule[-2 ex]{0ex}{0 ex} \\ \frac{1}{2(n+\kappa)} & \text{otherwise,} \end{cases}$$

with $i=0, \dots, 2n$. If $\underline{\boldsymbol{x}}$ follows a normal distribution, (Julier and Uhlmann 1997) suggest to use $\kappa = 3 - n$. For $n > 3$, however, $\kappa$ and the weight *w*_0_ of the first sigma point $\boldsymbol{x}_{0}$ will be negative. As a result, the estimated matrices are not guaranteed to be positive semi-definite and hence, covariance matrices (Julier 2002). Therefore, we empirically evaluated several choices for $\kappa > 0$. We found that $\kappa = 0.25$ performs best, which we will further use as scale factor within this work for the unscented transform.

We first calculate the transformed set of sigma points $\boldsymbol{y}_{i}$ to derive the transformed mean and covariance. In our case this transformation is the monoplotting where camera and ray are defined by each $\boldsymbol{x}_{i}$. The estimated mean $\boldsymbol{\mu}_{y}$ is then the weighted average of the transformed points

11 $$\boldsymbol{\mu}_{y} = \sum_{i=0}^{2n} w_{i}\boldsymbol{y}_{i}$$

with the estimated covariance matrix $\mathsf{\Sigma}_{yy}$ as

12 $$\mathsf{\Sigma}_{yy} = \sum_{i=0}^{2n} w_{i}(\boldsymbol{y}_{i}-\boldsymbol{\mu}_{y})(\boldsymbol{y}_{i}-\boldsymbol{\mu}_{y})^{\mathsf{T}} ,$$

which also is the estimate for $\mathsf{\Sigma}_{M\!M}$. As described in the beginning of Sec. 3.2, in our case, the random variable $\boldsymbol{\underline{x}}$ contains the orientation parameters and the image point, giving $n = 11$. Thus, 23 sigma points are necessary for this unscented transform, which is much less than for the Monte Carlo simulation.

#### Classical Variance Propagation

This approach requires a first-order approximation of the nonlinear expressions in Eq. 5, which is commonly done by truncating the Taylor series expansion after the first-order terms (Förstner and Wrobel 2016, p. 43). The derivation of the Jacobians with respect to the orientation parameters and the image point is given in Sec. 8.2. This approach is expected to be the fastest of all three uncertainty estimation methods, but at the cost of reduced accuracy of the estimated uncertainty due to the required approximation of the terrain.

### Silhouettes

So far, we discussed the three selected uncertainty estimation methods in more detail. Despite their different nature (i.e. sample based vs. variance propagation), all three approaches are influenced by silhouettes, which separate the visible terrain areas from the obstructed ones and are viewpoint dependent. As we represent the DTM using $C^{0}$ continuity, the location of silhouettes is limited to visible triangle edges with one obstructed neighbouring triangle (Hertzmann 1999).

Although silhouettes affect all three methods, the effects are different. For the classical variance propagation, the intersected triangle at ***M*** serves as the local tangent plane, which cannot represent the resulting depth discontinuities at the silhouettes. As a result, the uncertainty will be quite likely underestimated. In contrast, the intersected sample points $\boldsymbol{M}_{i}$ obtained during the Monte Carlo simulation and the unscented transform will generally be located on widely separated parts of the surface, thus forming several clusters. While, these two approaches capture the geometric situation caused by silhouettes, the derived “variances” (i.e. the main diagonal elements of $\mathsf{\Sigma}_{MM}$) cannot be interpreted as uncertainty measures because of the multimodal, and thus non-Gaussian, distribution of the intersection points $\boldsymbol{M}_{i}$.

Regardless of which method is used, the estimated monoplotting uncertainty at silhouettes is either expected to be underestimated or not statistically sound. Consequently, this situation should be identified either by masking the respective pixels in the uncertainty map (where the monoplotting uncertainty is estimated for every image pixel) or the user should get a warning (in case of manually picked points).

A special form of a silhouette is the horizon. There, some samples of the image rays will not intersect the terrain at all, in contrast to regular silhouettes. While this has no special consequences for the uncertainty estimation based on the variance propagation, the Monte Carlo simulation and unscented transform will be based on fewer intersected sample points $\boldsymbol{M}_{i}$. Depending on the number of lost samples, the accuracy of the estimates will decrease. Losing a few samples will have a much bigger effect on the unscented transform than on Monte Carlo. Accordingly, in the subsequent experiments, we limit our analyses only to those pixels where all sample points for the unscented transform intersect the terrain.

For each uncertainty estimation method, different intermediate results become available, which allow us to adapt the silhouette detection for each approach to optimally exploit the available information. Hence, in the following, we discuss the derivation of the silhouette masks for the different uncertainty estimation methods.

#### Monte Carlo Simulation

As outlined above, the modalities of the intersection points $\boldsymbol{M}_{i}$ offer a way to detect the vicinity of silhouettes. To test a one-dimensional distribution for unimodality, (Hartigan and Hartigan 1985) proposed the so-called dip test, where samples with resulting *p*-values $\leq 0.05$ can be considered bimodal (Freeman and Dale 2013; Elsen and Tingley 2015). Accordingly, we can derive a silhouette mask by transforming the 3D coordinates of the intersection points into a one-dimensional distribution and applying the dip test. For the transformation, we use

13 $$r_{i} = \frac{\overrightarrow{\boldsymbol{M}\boldsymbol{M}_{i}} \cdot \overrightarrow{\boldsymbol{Z}\boldsymbol{M}}}{|\overrightarrow{\boldsymbol{Z}\boldsymbol{M}}|}$$

where we project each intersected sample point $\boldsymbol{M}_{i}$ orthogonally onto the image ray of ***M***. We derive a one-dimensional distribution centred around zero by subtracting the distance between the projection centre ***Z*** and ***M***.

#### Unscented Transform

The same approach can be employed for the transformed sigma points from the unscented transform, but their count might not be sufficient for the dip test. However, we observed that the estimated mean $\boldsymbol{\mu}_{y}$ from the unscented transform Eq. 11 differs from the monoplotting point ***M*** in case the respective image pixel is in the vicinity of a silhouette. Accordingly, we use

14 $$s = \frac{|\boldsymbol{\mu}_{y} - \boldsymbol{M}|}{g_{M}},$$

for thresholding to find pixels in the vicinity of silhouettes. Here, *g*_*M*_ is the ground sampling distance of the pixel ***m*** calculated as

15 $$g_{M} = \frac{-\boldsymbol{c}_{3}^{\mathsf{T}}\cdot\overrightarrow{\boldsymbol{Z}\boldsymbol{M}}}{f},$$

where *f* is the principal distance and $\boldsymbol{c}_{3}$ the last column of the rotation matrix *R* (see Sec. 8.1), which is the opposite viewing direction. Dividing by *g*_*M*_ gives a relative measure, which can again be used for thresholding to derive the silhouette mask for the unscented transform.

#### Classical Variance Propagation

Classical variance propagation does not depend on sampling points, and hence, a different approach for silhouette detection is required. Accordingly, we aim to find them in image space by applying an edge detector to the depth map (Saito and Takahashi 1990). However, this cited approach requires a threshold that cannot be interpreted geometrically. To overcome this, we first derive the object coordinates ***M*** of each image pixel ***m*** by monoplotting. Subsequently, for each pixel $\boldsymbol{m}_{0}$ and its 8‑connected pixel neighbours $\boldsymbol{m}_{i}$ we calculate the Euclidean distances in object space between $\boldsymbol{M}_{0}$ and $\boldsymbol{M}_{i}$. From these 8 distances *d*_*i*_ per pixel, we calculate the ratio

16 $$\frac{\max(d_{i})}{\text{med}(d_{i})} \geq t_{1}$$

with $\text{med}(d_{i})$ being the median. In the vicinity of silhouettes, this ratio will become large, e.g. the maximum will be twice or three times larger than the median, whereas otherwise it will be close to 1. Thus, by thresholding with *t*_1_ we obtain a silhouette mask SM_0_ in image space. Up to this point, this mask only contains pixels directly at or close to the silhouettes. However, it does not account for distant pixels, which are affected by silhouettes just due to the uncertainty of their projection ray. To include these pixels as well, we use the monoplotting uncertainty estimated by variance propagation, which resembles an ellipse in the triangle plane of ***M***. Subsequently, we project the vertex and co-vertex of the 95% confidence ellipse from the object space into the image space. By that, we can calculate a value *t*_2_ for each pixel, which is the shorter of the projected semi-major and semi-minor axes. If the distance of an image pixel to the closest pixel of the silhouette mask SM_0_ is below *t*_2_, the respective pixel will also be masked. In the end, we arrive at the updated silhouette mask SM. As we anticipate that variance propagation will be used to derive the uncertainty map, which involves monoplotting and uncertainty estimation for all image pixels, no additional expensive calculations are required to derive the silhouette mask SM.

## Dataset Used for Evaluation

In the previous section, we discussed in detail the selected uncertainty estimation methods, explained how they are influenced by silhouettes and how we use the available information from the individual approaches to select pixels influenced by silhouettes. Especially, in Sec. 3.3 we discussed that no proper uncertainty estimates can be expected for points in the vicinity of silhouettes, independent of the employed method. This raises the question of how well we can detect and mask image pixels affected by silhouettes. Furthermore, the question remains how accurate the estimates based on variance propagation and unscented transform are compared to the Monte Carlo simulation for unaffected regions. We investigate these two questions in a real-world application using the historical image shown in Fig. 1:

1. In the first use case, we use the monoplotted glacier outline shown in Fig. 1. This is a typical example of how monoplotting is used in environmental sciences. For the monoplotted points, representing the vertices of the glacier outline, the estimated uncertainty will be directly interpreted by the user to decide if the results are useful, e.g. for investigating the glacier retreat over a certain timespan. Accordingly, the uncertainty estimates must be accurate.
2. The second use case addresses the uncertainty map, i.e. the estimation of the uncertainty for all image pixels. As mentioned, such a tool will be beneficial during image orientation to get an impression of the achievable monoplotting accuracy. Therefore, we anticipate that this map will be calculated multiple times for different settings and GCP constellations. Accordingly, its calculation should be fast, as otherwise its benefit gets lost.

Based on six GCPs (shown in Fig. 1) that were manually obtained from a recent orthophoto and DTM, the unknown camera parameters have been calculated by spatial resectioning in the software Oriental (Karel et al. 2013). The image measurements of the GCPs were equally weighted, and their object coordinates were fixed. Not only the parameters themselves but also their uncertainties were estimated: All elements of the exterior orientation ($X_{0},Y_{0},Z_{0},\alpha,\zeta,\kappa$) and the principal distance *f* for the interior orientation. Initially, the coordinates of the principal point were estimated as well. However, as those turned out to be too highly correlated with the rotation angles, they were fixed at the image centre. Consequently, for the unscented transform, 19 sigma points are required.

The estimated orientation parameters for the historical image are given in Table 1, and the coordinates of the GCPs in the image and the object coordinate system are listed in Table 2. If not otherwise mentioned, we use $\hat{\sigma}_{0} = 0.6~\text{px}$, estimated during the spatial resection, as image measurement uncertainty $\sigma_{m}$ for our monoplotting analysis.

**Table 1:** Camera orientation parameters for the historical image. Estimated camera parameters with their standard deviations in brackets.

*x*_0_ [px]	1000
*y*_0_ [px]	−665.5
$\hat{f}$ [px]	2200.1 (4.9)
$\hat{X}_{0}$ [m]	631,961.0 (1.7)
$\hat{Y}_{0}$ [m]	5,194,539.3 (1.4)
$\hat{Z}_{0}$ [m]	2169.6 (0.5)
$\hat{\alpha}$ [°]	−51.93 (0.03)
$\hat{\zeta}$ [°]	268.23 (0.03)
$\hat{\kappa}$ [°]	−89.47 (0.05)
$\hat{\sigma}_{0}$ [px]	0.6

**Table 2:** Coordinates of the GCPs for the historical image: Image ($x,y$) and object coordinates ($X,Y,Z$) in the UTM-32N coordinate system. The units are pixels and meters, respectively. The origin of the image system is the center of the top left pixel, with *x*-axis to the right, and *y*-axis up.

	*x*	*y*	*X*	*Y*	*Z*
2	410.8	−904.2	632,594.4	5,194,061.4	2108.8
4	1778.9	−819.5	632,279.4	5,193,591.3	2136.4
5	1228.2	−174.6	633,775.0	5,191,663.0	3040.9
7	383.9	−1087.8	632,460.0	5,194,170.6	2072.8
8	438.6	−198.3	636,614.9	5,190,978.5	3546.9
9	1251.2	−1031.3	632,432.6	5,193,770.2	2050.1

To represent the current topography, we used the publicly available DTM from the Land Tirol, which was created from recent Airborne Laser Scanning flights and has a resolution of $1~\text{m} \times 1~\text{m}$. We triangulated and simplified the DTM (Mandlburger et al. 2009) with the software OPALS (Pfeifer et al. 2014) to derive a mesh.

## Results

As throughout these experiments 1000 random rays are casted for the Monte Carlo simulation, we refer to it and its results as MC_1000_. Similarly, the results of the unscented transform are indicated by UT_19_, as it is based on 19 sigma points. Finally, the approach and the results obtained by classical variance propagation and the local tangential plane of the monoplotting point will be further addressed by TANG.

To compare the results from the different uncertainty estimation methods, we derive two common measures from $\mathsf{\Sigma}_{MM}$ to individually represent the planimetric and height uncertainty of each monoplotted point ***M***. For the planimetric uncertainty, further referred to as $\sigma_{\text{2D}}$, we use Helmert’s position error calculated as

17 $$\sigma_{\text{2D}} = \sqrt{\sigma^{2}_{X} + \sigma^{2}_{Y}}$$

and for the height uncertainty, we directly use

18 $$\sigma_{\text{H}} = \sqrt{\sigma_{Z}^{2}}.$$

Here, $\sigma^{2}_{X}$, $\sigma^{2}_{Y}$ and $\sigma^{2}_{Z}$ are the variances of the object coordinates, which make up the main diagonal of the covariance matrix $\mathsf{\Sigma}_{MM}$.

We first discuss the quality of the derived silhouette masks (Sect. 5.1), before evaluating the uncertainty estimation for individual monoplotted points (Sect. 5.2) and the whole image (Sect. 5.3).

### Silhouette Masks

As reference for evaluating the derived silhouette masks, we use the mask based on the 1000 Monte Carlo sampling points, further referred to as MC_DIP. As mentioned in Sec. 3.3, we test the distribution of the monoplotted sampling points for unimodality with the dip test (Hartigan and Hartigan 1985) based on Eq. 13 and use a threshold of 0.05 as suggested by (Freeman and Dale 2013; Elsen and Tingley 2015). The same threshold is also used to derive the UT_DIP mask, using the 19 sigma points from the unscented transform within the dip test. Since we anticipate that the 19 samples for the dip test will not be enough, we additionally derive a second mask for the unscented transform based on Eq. 14. This mask will be labelled as UT_DIST and uses a threshold of 0.4. The mask for the classical variance propagation is derived by the local distance ratio Eq. 16, for which we empirically found a threshold of 2.2 worked best. This mask SM_0_ is then further padded as described in Sect. 3.3.3 to derive the mask TANG_DIST. Note, while for the masks based on the dip test we followed (Hartigan and Hartigan 1985) and (Freeman and Dale 2013) for the selection of the threshold, in the other two cases we evaluated several thresholds and selected for each approach one which resulted in a higher recall at the cost of a lower precision.

**Fig. 2:**
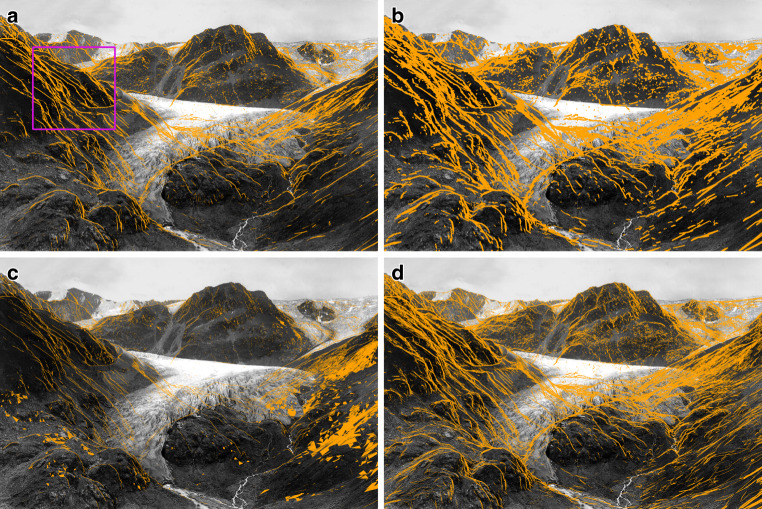
Silhouette masks derived from the different detection approaches. The purple square indicates the enlarged area shown in Fig. 3. **a** MC_DIP—Dip test for the 1000 Monte Carlo sampling rays. **b** TANG_DIST—Distance ratio of neighbouring image pixels. **c** UT_DIP—Dip test for the 19 unscented transform sigma points. **d** UT_DIST—Distance between $\boldsymbol{\mu}_{\boldsymbol{y}}$ and *M*

Figure 2 compares the obtained masks visually, Table 3 lists precision, recall and Matthews correlation coefficient (MCC) (Matthews 1975) of the different methods and for two different image measurement uncertainties $\sigma_{m}$.

**Table 3:** Precision (PREC), recall (REC) and Matthews correlation coefficient (MCC) for the three silhouette masks for two different image measurement uncertainties $\sigma_{m}$. PREC and REC are reported as percentages. As reference, the MC_DIP mask is used.

	$\sigma_{m}=\hat{\sigma}_{0}=0.6~\text{px}$			$\sigma_{m}=2.2~\text{px}$		
	PREC	REC	MCC	PREC	REC	MCC
UT_DIP	50.3	35.2	0.35	73.0	15.2	0.27
UT_DIST	48.9	85.0	0.58	39.6	87.8	0.43
TANG_DIST	43.2	93.4	0.57	42.8	97.3	0.52

From Fig. 2, one can directly see that all derived silhouette masks basically capture the same structures. While UT_DIST appears very similar to the reference MC_DIP, the extracted structures in TANG_DIST are broader. UT_DIP generally differs to a greater degree. First, the extracted structures are much thinner. Second, whole triangles in the right part of the image are masked. This shows that using the 19 sigma points for the dip test is only sufficient in the direct proximity of the silhouettes and in areas unaffected by them. However, this approach cannot capture the influence of silhouettes on pixels further away. In contrast, using UT_DIST, based on the distance between the unscented transform mean $\boldsymbol{\mu}_{y}$ and ***M***, appears as a promising alternative.

**Fig. 3:**
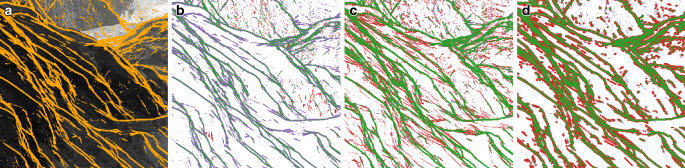
Comparison of the silhouette masks. Green pixels indicate correctly classified silhouette pixels (true positives), purple pixels show false negatives, and red pixels show false positives. **a** MC_DIP (Reference), **b** UT_DIP, **c** UT_DIST, **d** TANG_DIST

To discuss this in more detail, the results from a small region of the original image, depicted as a purple square in Fig. 2, are shown in Fig. 3. Pixels in green indicate correct classifications, whereas purple pixels indicate false negatives, and red pixels represent false positives. This further confirms our observation for the silhouette mask derived from the dip test of the 19 sigma points (UT_DIP). While the pixels directly at the silhouettes are correctly classified, all others are missed. Accordingly, this approach achieves a higher precision (Table 3—left) but very low recall and hence, is less useful as a mask. In contrast, the masks from UT_DIST and TANG_DIST perform very similarly: The major structures and their width are detected very well, and only minor structures are missed. Accordingly, also their precision (48.9% vs. 43.2%), recall (85.0% vs 93.4%) and MCC score (0.58 vs. 0.57) are very similar (Table 3—left).

The quality of the derived masks for a larger image measurement uncertainty of $\sigma_{m}=2.2~\text{px}$ (to indicate a worse identification accuracy of object points of interest in contrast to the original GCPs that define the $\hat{\sigma}_{0}$) are shown in Table 3 (right). This indicates that the achieved scores for all three methods are similar for both $\sigma_{m}$.

These results demonstrate, that using the available information from both uncertainty estimation methods (i.e. UT_19_ and TANG) allows us to derive silhouette masks with a high recall, which are independent of the image measurement uncertainty.

### Uncertainty of Monoplotted Points

**Fig. 4:**
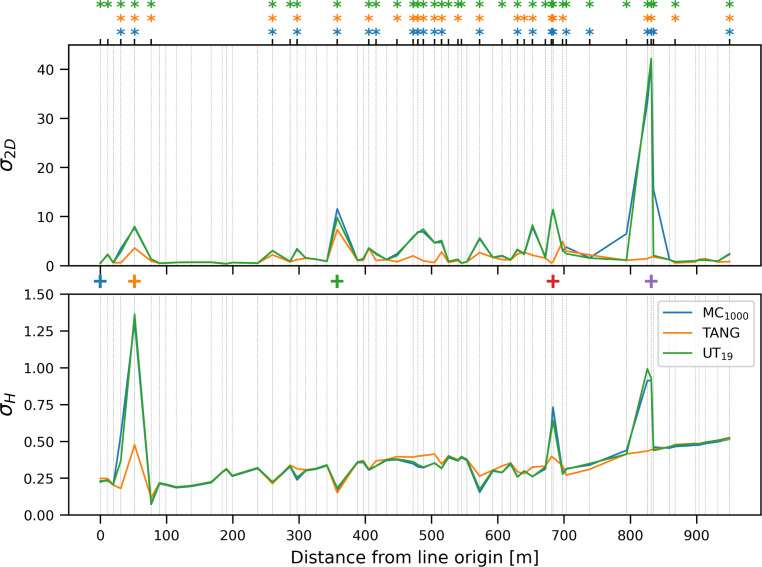
The estimated uncertainties $\sigma_{\text{2D}}$ (top) and $\sigma_{\text{H}}$ (bottom) for the vertices of the monoplotted line in Fig. 1. The coloured plus sign (+) between both plots corresponds to the coloured markers in Fig. 1. Accordingly, the blue marker represents the line origin in object space in Fig. 1. The asterisks on top indicate vertices which would be masked by MC_DIP (blue), UT_DIST (orange) and TANG_DIST (green)

We estimated the uncertainties of the 61 vertices of the monoplotted line representing the glacier tongue in Fig. 1 using MC_1000_, UT_19_ and TANG. From Fig. 4 one can see, that for most vertices the planimetric uncertainty varies within 0 and 10 m, except for one region at distance 100 to 250 m from the line origin where the uncertainties stay close to zero and one vertex with a huge planimetric uncertainty of $\sim40~\text{m}$ around 800 m. This already shows the variability of the uncertainties of the individual vertices of a line, which itself is already an interesting observation and proves that estimating the uncertainty for each *vertex* provides us with a much more differentiated possibility to discuss the uncertainty of the monoplotted *line*.

Overall, the height uncertainty is significantly smaller than the planimetric uncertainty. For most vertices, it is around 0.25 m and varies to a much lesser degree. This is due to the oblique viewing geometry, where small uncertainties of the projection ray lead to larger changes in the planimetric coordinates.

Regarding the three estimation methods, the results from MC_1000_ and UT_19_ are nearly identical, whereas the TANG estimates are different. The TANG results are generally smaller for $\sigma_{\text{2D}}$, whereas for $\sigma_{\text{H}}$, over- and underestimations happen almost equally. These differences occur close to silhouettes (indicated by the asterisks at the top of Fig. 4) and are caused by the tangent plane approximation of the DTM in the monoplotted point ***M***. Especially for $\sigma_{\text{2D}}$, it is easy to see that the tangent plane “keeps” the variation close to M, whereas MC_1000_ and UT_19_ consider ray variations that intersect the DTM also further away.

To quantitatively analyse these observations, we calculate the vertex-wise relative differences between each approach and MC_1000_ as

19 $$\frac{\sigma_{\text{2D}} - \sigma_{\text{2D},\text{MC}_{1000}}}{\sigma_{\text{2D},\text{MC}_{1000}}} 100.$$

This measure is further referred to as relative difference and is reported for the vertices of the glacier outline with and without masking in Table 4. The obtained values further confirm our observations. The TANG approach clearly benefits from considering the silhouette mask, which is expected, as the TANG estimates at silhouettes are bound to be very wrong. However, for UT_19_, these relative differences are much smaller, indicating that its results are closer to the reference, even in the vicinity of silhouettes. Overall, UT_19_ is significantly more accurate, masked and unmasked. As the estimated uncertainties of the individual vertices will be directly used and interpreted by the user, our initial idea to use either MC_1000_ or UT_19_ for the uncertainty estimation of individual, hand-picked, vertices is further confirmed. However, for vertices close to silhouettes, a warning should be triggered for the user.

**Table 4:** Relative differences Eq. 19 of $\sigma_{\text{2D}}$ for the different approaches for the vertices of the glacier outline. STD is the standard deviation, and RMS is the root mean square error. The reference mask MC_DIP was applied to calculate the masked metrics.

Method	Mean [%]	STD [%]	RMS [%]
TANG	−26.1	37.6	45.8
TANG (masked)	−6.6	23.8	24.7
UT_19_	−3.2	16.6	16.9
UT_19_ (masked)	−1.9	14.0	14.1

### Uncertainty Map

**Fig. 5:**
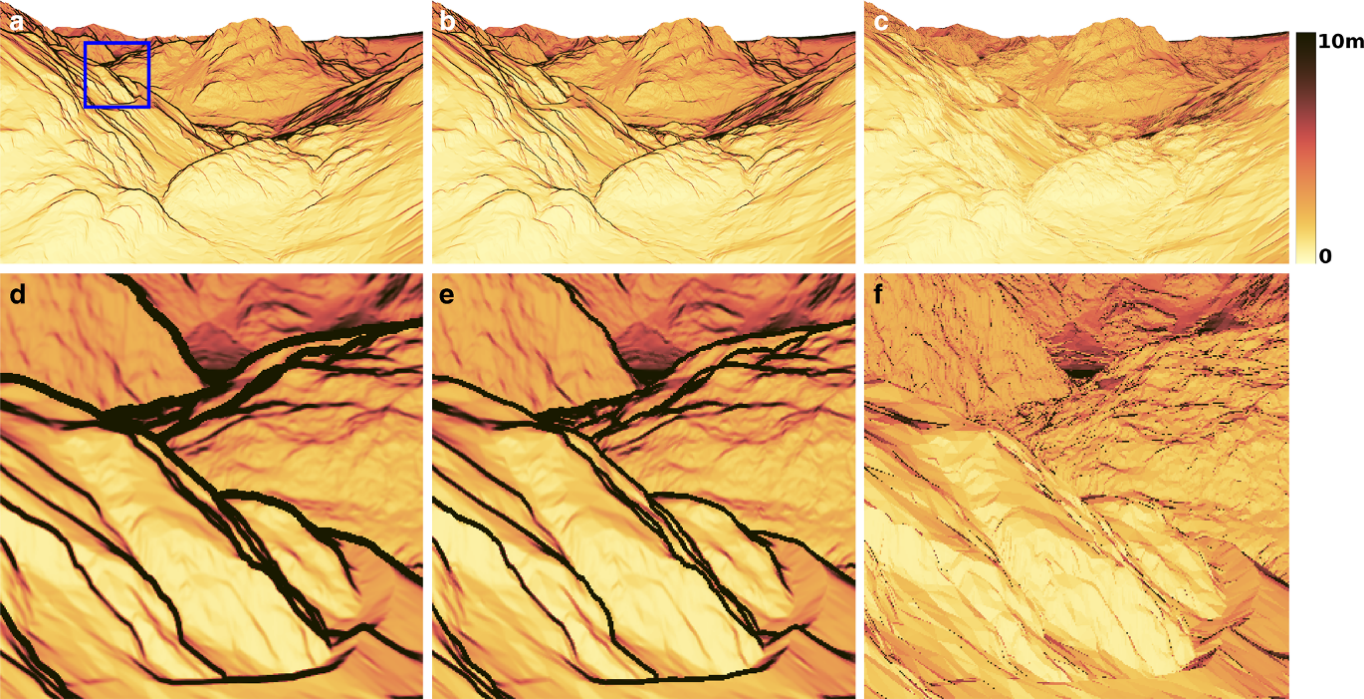
Estimated planimetric uncertainty $\sigma_{\text{2D}}$. The top row shows the whole image, and the bottom row shows the area within the blue square. For visual comparison, the silhouettes have not been masked. **a** MC_1000_ (Reference), **b** UT_19_, **c** TANG, **d** MC_1000_ (Reference), **e** UT_19_, **f** TANG

For the derivation of the uncertainty map, we estimated the monoplotting uncertainty for each pixel of the selected historical image using MC_1000_, UT_19_ and TANG (Fig. 5). In contrast to the previous evaluation for the glacier outline, we restrict the discussion to $\sigma_{\text{2D}}$. We use the UT_DIST mask for UT_19_, and the mask derived by TANG_DIST for TANG.

It is immediately apparent from Fig. 5 that the TANG estimates in the vicinity of the silhouettes, as expected, are significantly smaller than the MC_1000_ reference and UT_19_. While the values in the remaining areas appear similar, the results from both MC_1000_ and UT_19_ look blurred compared to TANG, where the triangles become visible. Both MC_1000_ and UT_19_ are based on the actual shape of the terrain. Therefore, the sampling points for each image pixel will also lie on different triangles (especially for monoplotted points close to triangle edges). This results in slightly different uncertainty estimates for neighbouring pixels, like being obtained by a moving average window, whose diameter is defined by the spread of the sampled rays. In contrast, TANG is based on the local tangential plane. All monoplotted points within the same triangle will therefore use the same tangent plane for the estimation of the uncertainty, no matter how far or close they are to the triangle edges.

**Fig. 6:**
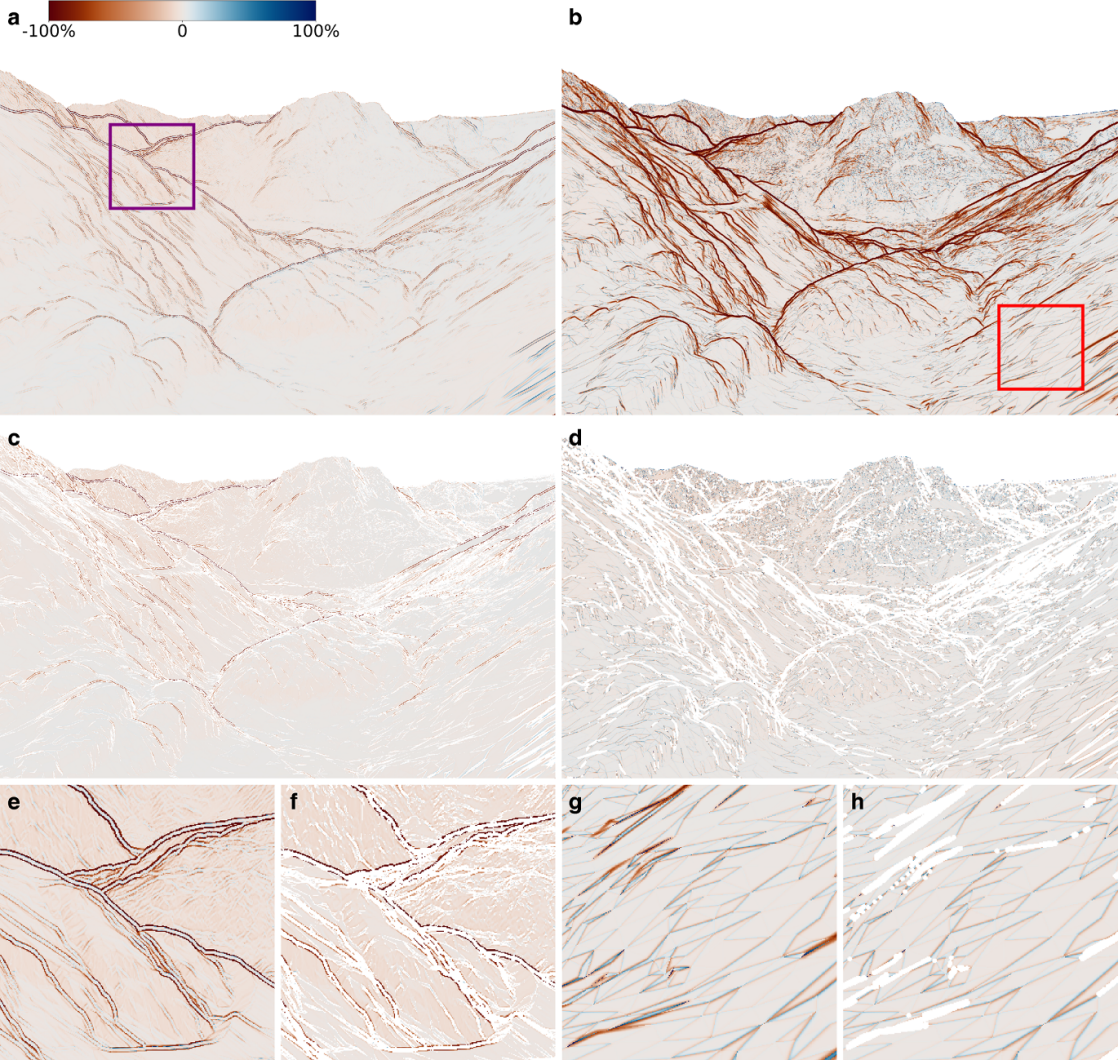
Relative pixel wise differences of $\sigma_{\text{2D}}$ calculated with Eq. 19 in image space. **a**,**b** show the whole image unmasked, **c**,**d** masked. **e**,**f** show the area within the purple square, **g**,**h** show the area within the red square. For the visualisation, we clipped the values to $[-100\%, +100\%]$. **a** UT_19_, **b** TANG, **c** UT_19_ with UT_DIST, **d** TANG with TANG_DIST, **e** UT_19_, **f** UT_19_ with UT_DIST, **g** TANG, **h** TANG with TANG_DIST

These observations are further confirmed in Fig. 6, which shows the relative differences Eq. 19 in $\sigma_{\text{2D}}$ of all image pixels, with red areas representing regions where MC_1000_ is larger than the respective approach. From Fig. 6a,b, it is evident that the main differences indeed occur along the silhouettes, and with the silhouette masks (Fig. 6c,d), the results look very similar. Two interesting patterns can be observed.

First, although UT_19_ generally produces very accurate results, double lines of wrong estimates parallel to silhouettes are visible (Fig. 6e,f). This is related to the selected scaling factor $\kappa$ of 0.25, which leads to correct estimates within a narrow band centred along the silhouettes. At both edges of this band, however, $\kappa$ is too small, so that the sigma points are no longer on both sides of the silhouettes, although they should be. This leads to this overoptimistic symmetrical pattern.

Second, for TANG, the largest remaining relative differences in the masked difference image appear close to the edges of the triangles (Fig. 6g,h), where under- and overestimation occur on both sides of the edges. Consequently, one can again identify the triangular structure of the underlying DTM, now even more pronounced than in Fig. 5f. As already discussed above, this is related to the difference in how these two approaches estimate the uncertainties, which leads to an under- and overestimation on both sides of the edges for TANG.

**Fig. 7:**
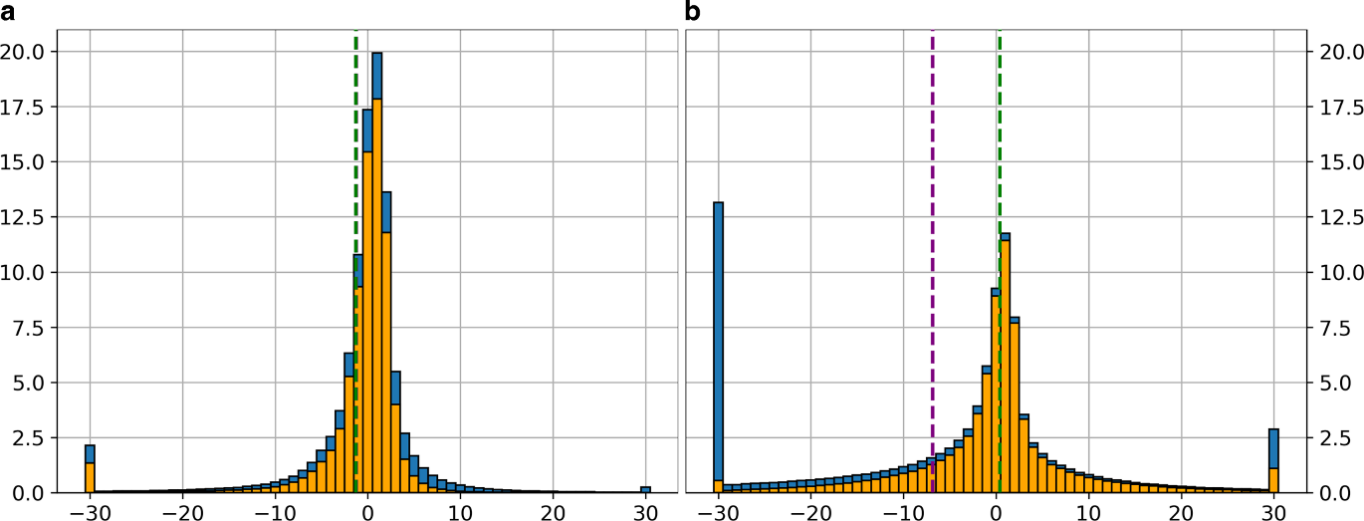
Histograms of the relative frequencies of the relative differences in $\sigma_{\text{2D}}$ Eq. 19 of all image pixels, both unmasked (blue) and masked (orange). The histograms have a bin width of 1% and the differences are clipped to $\pm30\%$. The relative frequencies were obtained by dividing by the same pixel count. The mean of the relative differences for the unmasked images is indicated as the purple dashed line, and for the masked images, it is indicated as the green dashed line. For UT_19_, both means are equal. **a** Relative frequencies for UT_19_ both masked (orange) and unmasked (blue). **b** Relative frequencies for TANG both masked (orange) and unmasked (blue)

For quantitative analyses, we show the histograms of the relative frequencies of the relative differences in $\sigma_{\text{2D}}$ Eq. 19 of all image pixels, both unmasked (blue) and masked (orange) in Fig. 7. Statistical measures are listed in Table 5.

**Table 5:** Relative differences of $\sigma_{\text{2D}}$ Eq. 19 for the different approaches considering all pixels (top) and only pixels within $\pm 30\%$. STD is the standard deviation, and RMS is the root mean square error. The column “valid” refers to the number of pixels considered for the respective calculation in relation to all pixels of the image. Values highlighted with * are the best results.

	Method	Valid [%]	Mean [%]	STD [%]	RMS [%]
All	TANG	100.0	−6.9	52.8	53.3
	TANG (masked)	73.6	0.4*	43.5	43.5
	UT_19_	100.0	−1.3	11.6	11.7
	UT_19_ (masked)	78.8	−1.3	9.4*	9.5*
$\pm 30\%$	TANG	84.2	−1.3	9.4	9.5
	TANG (masked)	72.0	−0.2	7.8	7.8
	UT_19_	97.7	0.0*	4.6	4.6
	UT_19_ (masked)	77.4	−0.2	3.5*	3.5*

Most of the original (i.e. unmasked) differences are close to zero for both approaches. However, the percentage of outliers is significantly smaller ($\sim 2\%$) for UT_19_ than for TANG ($\sim 15\%$). For TANG, the majority ($\sim 12.5\%$) is negative, which are the pixels in the vicinity of the silhouettes, where TANG underestimates the uncertainty (as expected). With the mask, the number of pixels with extreme differences drops below $\sim 1\%$ for TANG, whereas for UT_19_ the silhouette mask influences all regions of the histogram equally.

Overall, the number of pixels within $\pm 5\%$ is significantly larger for UT_19_, which shows that UT_19_ is more accurate than TANG around the silhouettes and the remaining parts of the whole image. The same was observed for the vertices of the glacier outline in Sect. 5.2 and Fig. 4. This is further confirmed by calculating the standard deviation (STD) and root mean square (RMS) of the relative differences (Table 5). Without the mask, the STD and RMS for TANG are $\sim 53\%$, which is reduced to $43.5\%$ when applying the mask. However, for UT_19_ both metrics are $\sim 12\%$ without the mask and $\sim 9.5\%$ with the mask. Masked or unmasked, UT_19_ is always closer to the Monte Carlo reference.

The reason for this is that the magnitude of the remaining outliers for masked TANG is much larger than for the outliers in the masked UT_19_, while their number is similar (see the overflow bars of the masked histogram in Fig. 7). Therefore, we limit the calculation of the metrics to the pixels in the visually reliable range $[-30\%, +30\%]$, to derive representative quality measures (Table 5—bottom). Now, the STD and RMS become $\sim 9.5\%$ (unmasked) and $\sim 8\%$ (masked) for TANG, and 4.6% (unmasked) and 3.5% (masked) for UT_19_. While these values are still a bit better for UT_19_ than for TANG, the values for TANG are not much worse. Since the uncertainty map is anticipated to be derived by the TANG method, these latter values indicate the quality of the uncertainty map in regions not affected by silhouettes.

### Runtime Comparison

So far we showed, that the results from UT_19_ are closer to MC_1000_ than for TANG in both anticipated use cases. However, besides their accuracy also the runtime of the individual approaches further influences their practicability. Accordingly, we measured the runtime for computing the uncertainty map for each approach, which is listed in Table 6. In total, MC_1000_ requires $\sim$ 33 min, UT_19_ $\sim$ 6 min and TANG only 4 s. The average runtime per pixel (last row in Table 6) shows that each approach is faster than one millisecond. For the ray casting, we used the Python library Open3D (Zhou et al. 2018), which employs Embree for the ray casting in the background. As such, the ray casting itself is highly optimised in C++, whereas the covariance estimation was implemented purely in Python.

**Table 6:** Runtime of the individual uncertainty estimation methods in seconds, which is split into the time required for the ray casting and covariance derivation. The first three rows show the runtime for the whole image ($2.6\times 10^{6}~\text{pixels}$), the last row indicates the average runtime per pixel.

	MC_1000_	UT_19_	TANG
Ray casting	1529	25	1
Covariance	491	352	3
Total	2020	377	4
$\varnothing~\text{per pixel}$	$7.5\times 10^{-4}$	$1.4\times 10^{-4}$	$1.5\times 10^{-6}$

For MC_1000_, most of the time is required for intersecting the 1000 random rays per pixel. In case of UT_19_, the subsequent calculation of Eqs. 11 and 12 takes 14 times longer than the ray casting itself, which is partly because these calculations have not been optimised. In contrast, TANG was implemented using vectorised functions in NumPy (Harris et al. 2020), thus avoiding the notoriously slow for-loops in Python. Still, calculating the covariance matrices takes 3 times as long as the ray casting. Nonetheless, the whole workflow for TANG is 6 times faster than only the ray casting for UT_19_.

## Discussion

Given our results, estimating the monoplotting uncertainty of individual points by UT_19_ is an accurate alternative to MC_1000_. With UT_19_, quite precise estimates for the monoplotting uncertainty are obtained for our glacier outline example (with an RMS of 14.1%), which is important for the subsequent (e.g. geomorphological) analyses. And considering that these points are manually identified, the time required for ray casting 18 additional points for the UT_19_ uncertainty estimates is of no concern. However, both MC_1000_ and UT_19_ are fast enough ($7.5\times 10^{-4}$ vs. $1.4\times 10^{-4}$ s per pixel) to integrate them into the monoplotting workflow. While their runtime difference is negligible for individual points, using MC_1000_ has various advantages: First, MC_1000_ is the most accurate approach. Second, opposite to the unscented transform, no $\kappa$ value needs to be selected. Thus, removing one open issue, because the $\kappa$ value, found for our example, still would need to stand the test of applicability onto other images. And third, using the dip test, pixels affected by silhouettes can be detected easily. Therefore, despite the nominally longer runtime compared to the unscented transform, we suggest using the Monte Carlo simulation for the uncertainty estimation of individual points.

In contrast, the uncertainty map aims to provide the user with an estimate of the expectable monoplotting uncertainties. As motivated earlier, this can be particularly helpful during the orientation of the images as it might help to decide whether further time needs to be spent on selecting additional GCPs. Accordingly, this uncertainty map might be calculated multiple times during image orientation. Therefore, it is important that this map can be derived relatively fast—at the same time, the values in the uncertainty map need not be of utmost precision.

Consequently, this example supports our initial idea to use the TANG approach for deriving the uncertainty map together with the silhouette mask. It requires a single ray casting per pixel, other than UT_19_, which requires 19. This makes a huge difference for the whole image with 2.6 million pixels, as can be seen from the runtime for the ray casting alone (25 vs 1 s). With our Python implementation for the subsequent covariance estimations, the TANG method derives the uncertainty map in a total of 4 s, which is 6 times faster than the ray casting for UT_19_ alone. Since the regions close to the silhouettes will be affected with larger errors anyway, these regions (and the actual obtainable uncertainties there) are of lesser interest during GCP selection for the orientation. Instead, the focus should be on areas away from the silhouettes where the TANG-based uncertainty map for our test image allows for reasonable predictions for the obtainable monoplotting uncertainty (with an RMS of 7.8%).

While we only used one image for the evaluation, various geometrical situations (e.g. incidence angle, normal vector, distance, triangle size), especially for the creation of the uncertainty map, are present. Accordingly, we believe that our results are generally representative.

## Conclusion

We considered the problem of estimating the uncertainty of monoplotted points. For this, we compared three approaches: i) unscented transform with 19 systematically selected rays (UT_19_), ii) first-order variance propagation with a tangential plane approximation offering a direct solution (TANG) and iii) Monte Carlo simulation with 1000 random rays (MC_1000_), which also served as reference. For the evaluation, one historical image was used, and we particularly addressed two different use cases: First, the monoplotting of individual, manually selected, image points, in our example representing a glacier outline, and secondly, the estimation of the uncertainty for each pixel of the image, referred to as the uncertainty map.

As the uncertainty estimation in vicinity of the silhouettes will not be statistically sound (MC_1000_, UT_19_) or will not be based on the true geometrical situation and hence, tends to an underestimation of the uncertainty (TANG), we also evaluated the derivation of silhouette masks for each uncertainty estimation method. For Monte Carlo simulation and unscented transform, we applied the dip test, proposed by (Hartigan and Hartigan 1985), to detect multimodal clusters of the intersected sampling points. As the dip test only works for one-dimensional distributions, we first transformed the 3D coordinates of the sampling points using Eq. 13. This showed that the 19 sigma points used for the unscented transform are not sufficient within this test to derive an accurate silhouette mask (UT_DIP). As an alternative, we calculated the distance between the estimated mean from the unscented transform ($\boldsymbol{\mu}_{\boldsymbol{y}}$) and the monoplotted point (***M***) itself in Eq. 14, which proved to be a better measure for deriving an accurate mask (UT_DIST). As TANG is not based on sampling rays, the dip test could not be applied. Therefore, we derived the silhouette mask based on the neighbourhood distance ratio Eq. 16 in object space (TANG_DIST) with an added step to include the stochasticity by mapping the 3D confidence ellipsoid into the image space. Evaluation showed that with both UT_DIST and TANG_DIST we achieved a high recall ($\sim 90\%$) with medium precision ($\sim 50\%$).

For the estimation of the monoplotting uncertainty of the individual, manually selected, vertices of a glacier outline, the estimates for UT_19_ (14.1% RMS) were more accurate than for TANG (24.7% RMS). However, as the estimation of the uncertainty using MC_1000_ is fast enough to be incorporated into the monoplotting workflow, MC_1000_ (with a theoretical precision of 2%) should be the chosen method for this use case. This is further supported by two additional criteria: First, the pixels affected by silhouettes can be detected more reliably using Eq. 13 than for UT_19_. And second, selecting the $\kappa$ coefficient is not necessary for MC_1000_.

For the derivation of the uncertainty map, similar results to those for the glacier outline were achieved. Excluding the area around the silhouettes, the tangential approximation is able to estimate the uncertainties with a relative RMS of 9.5%. The unscented transform, with $\kappa=0.25$, is very close to the Monte Carlo reference, yielding a relative RMS of 3.5%. However, in contrast to individual linear features with tens to hundreds of vertices, whole images consist of millions of pixels. Accordingly, the cost of 18 additional ray castings per pixel is not negligible for whole images (25 s for UT_19_ vs. 1 s for TANG). Because of this efficiency, we conclude that the tangential approach should be used for the uncertainty map. This way, creating the uncertainty map took 4 s in total.

Although the investigation emerged from our work with historical terrestrial images, we believe that the outcome will be of interest for the general application of monoplotting.

## Data Availability

The DTM for Kaunertal (Tirol) can be downloaded from https://www.tirol.gv.at/sicherheit/geoinformation/geodaten-tiris/laserscandaten/.

## Appendix

### Rotation Matrix $\textbf{\textsf{R}}_{\boldsymbol{\alpha\zeta\kappa}}$

Since this work focuses on terrestrial images, which are characterised by having a more horizontal than vertical viewing direction, we choose the $\alpha\zeta\kappa$-parametrisation, which is e.g. provided in the software Oriental (Karel et al. 2013). The rotation matrix $\textit{\textsf{R}}_{\alpha\zeta\kappa}$ is

20 $$\begin{aligned} & \textit{\textsf{R}}_{\alpha\zeta\kappa} = \left[\begin{array}{c c c} \cos{\alpha} & -\sin{\alpha} & 0 \\ \sin{\alpha} & \cos{\alpha} & 0 \\ 0 & 0 & 1 \\ \end{array} \right] \left[ \begin{array}{c c c} \cos{\zeta} & 0 & \sin{\zeta} \\ 0 & 1 & 0 \\ -\sin{\zeta} & 0 & \cos{\zeta} \\ \end{array} \right] \left[ \begin{array}{c c c} \cos{\kappa} & -\sin{\kappa} & 0 \\ \sin{\kappa} & \cos{\kappa} & 0 \\ 0 & 0 & 1 \\ \end{array} \right] \end{aligned}$$

21 $$ \phantom{\textit{\textsf{R}}_{\alpha\zeta\kappa}\,}= \left[ \begin{array}{c|c|c} \cos{\alpha}\cdot \cos{\zeta} \cdot \cos{\kappa} - \sin{\alpha}\cdot \sin{\kappa} & -\cos{\alpha}\cdot \cos{\zeta} \cdot \sin{\kappa} - \sin{\alpha}\cdot \cos{\kappa} & \cos{\alpha}\cdot \sin{\zeta} \\ \sin{\alpha}\cdot \cos{\zeta} \cdot \cos{\kappa} + \cos{\alpha} \cdot \sin{\kappa} & -\sin{\alpha}\cdot \cos{\zeta}\cdot \sin{\kappa} + \cos{\alpha}\cdot \cos{\kappa} & \sin{\alpha}\cdot \sin{\zeta} \\ -\sin{\zeta}\cdot \cos{\kappa} & \sin{\zeta}\cdot \sin{\kappa} & \cos{\zeta} \end{array} \right],$$

where $\alpha$ is the direction of the camera relative to the east, $\zeta$ the inclination of the camera relative to the nadir and $\kappa$ the rotation around the viewing axis.

### Derivation of the Jacobians

For the ray-plane intersection Eq. 5, the matrix of the first derivatives *J* is a $3\times11$ matrix: Three rows because the intersection point ***M*** is 3‑dimensional. Eleven columns because we have eleven random variables, namely the coordinates of the projection centre ***Z***, the Euler angles ($\alpha,\zeta,\kappa$), the interior orientation $\boldsymbol{z} = (x_{0},y_{0},f)^{\mathsf{T}}$ and the image coordinates $\boldsymbol{m} = (x_{m},y_{m},0)^{\mathsf{T}}$. Hence, *J* is made up of six sub-matrices

22 $$\underset{3\times 11}{\textit{\textsf{J}}} = \left[ \begin{array}{c|c|c|c|c|c} \underset{3\times 3}{\textit{\textsf{J}}_{MZ}} & \underset{3\times 1}{\textit{\textsf{J}}_{M\alpha}} & \underset{3\times 1}{\textit{\textsf{J}}_{M\zeta}} & \underset{3\times 1}{\textit{\textsf{J}}_{M\kappa}} & \underset{3\times 3}{\textit{\textsf{J}}_{Mz}} & \underset{3\times 2}{\textit{\textsf{J}}_{Mm}} \end{array} \right].$$

By combining these 11 parameters in the vector $\boldsymbol{g} = (\boldsymbol{Z}^{\mathsf{T}},~\alpha,~\zeta,~\kappa,~\boldsymbol{z}^{\mathsf{T}},~\boldsymbol{m}^{\mathsf{T}})^{\mathsf{T}}$ the differential change d***M*** in the intersection point ***M*** due to differential changes d***g*** in these parameters would be obtained by

23 $$\mathrm{d}\boldsymbol{M} = \textit{\textsf{J}} \mathrm{d}\boldsymbol{g} .$$

The partial derivatives of ***M*** with respect to the coordinates of the projection centre ***Z*** are

24 $$\textit{\textsf{J}}_{MZ} = \frac{\partial \boldsymbol{M}}{\partial \boldsymbol{Z}} = \textit{\textsf{I}}_{3} - \frac{\boldsymbol{d}\,\boldsymbol{n}^{\mathsf{T}}}{\boldsymbol{n}^{\mathsf{T}} \boldsymbol{d}} .$$

Using the partial derivatives with respect to the direction vector ***d***

25 $$\textit{\textsf{J}}_{Md} = \frac{\partial \boldsymbol{M}}{\partial \boldsymbol{d}} = \dfrac{\boldsymbol{n}^{\mathsf{T}} (\boldsymbol{P}_{0}-\boldsymbol{Z})}{\boldsymbol{n}^{\mathsf{T}} \boldsymbol{d}} (\textit{\textsf{I}}_{3} - \frac{\boldsymbol{d}\,\boldsymbol{n}^{\mathsf{T}}}{\boldsymbol{n}^{\mathsf{T}} \boldsymbol{d}}) = \lambda \textit{\textsf{J}}_{MZ}$$

the partial derivatives with respect to the Euler angles become

26 $$\textit{\textsf{J}}_{M\alpha} = \frac{\partial \boldsymbol{M}}{\partial \alpha} = \textit{\textsf{J}}_{Md} \begin{bmatrix} -\boldsymbol{r_{2}}^{\mathsf{T}} \\ \hspace{1em} \boldsymbol{r_{1}}^{\mathsf{T}} \\ \hspace{1.3em} \boldsymbol{0}^{\mathsf{T}} \end{bmatrix} (\boldsymbol{m}-\boldsymbol{z})$$

27 $$\textit{\textsf{J}}_{M\zeta} = \frac{\partial \boldsymbol{M}}{\partial \zeta} = \textit{\textsf{J}}_{Md} \begin{bmatrix} \boldsymbol{r}_{3}^{\mathsf{T}} \cos{\alpha} \\ \boldsymbol{r}_{3}^{\mathsf{T}} \sin{\alpha} \\ -\boldsymbol{r}_{1}^{\mathsf{T}} \cos{\alpha} - \boldsymbol{r}_{2}^{\mathsf{T}} \sin{\alpha} \end{bmatrix} (\boldsymbol{m}-\boldsymbol{z})$$

28 $$\textit{\textsf{J}}_{M\kappa} = \frac{\partial \boldsymbol{M}}{\partial \kappa} = \textit{\textsf{J}}_{Md} \begin{bmatrix} \boldsymbol{c_{2}} & -\boldsymbol{c_{1}} & \boldsymbol{0} \end{bmatrix} (\boldsymbol{m}-\boldsymbol{z})$$

with the rows and columns of *R* as

29 $$\textit{\textsf{R}} = \begin{bmatrix} \boldsymbol{r}_{1}^{\mathsf{T}} \\ \boldsymbol{r}_{2}^{\mathsf{T}} \\ \boldsymbol{r}_{3}^{\mathsf{T}} \end{bmatrix} = \begin{bmatrix} \boldsymbol{c}_{1} & \boldsymbol{c}_{2} & \boldsymbol{c}_{3} \end{bmatrix}.$$

For the interior orientation, the partial derivatives

30 $$\textsf{\textsl{J}}_{Mz} = \frac{\partial \boldsymbol{M}}{\partial \boldsymbol{z}} = -\textsf{\textsl{J}}_{Md}\textsf{\textsl{R}}$$

and finally, the partial derivatives for the image coordinates

31 $$\textit{\textsf{J}}_{Mm} = \frac{\partial \boldsymbol{M}}{\partial \boldsymbol{m}} = \textit{\textsf{J}}_{Md} \begin{bmatrix} \boldsymbol{c}_{1} & \boldsymbol{c}_{2} \end{bmatrix}.$$

## Code Availability

The uncertainty propagation will be integrated into our monoplotting software moniQue (https://github.com/smfloery/moniQue), which was realised as a QGIS plugin.
